# 基于通过型固相萃取-超高效液相色谱-串联质谱法同时测定牛蛙中9种雌激素

**DOI:** 10.3724/SP.J.1123.2022.01017

**Published:** 2022-07-08

**Authors:** Qiaoli QIU, Xiaohong CHEN, Shengdong PAN, Micong JIN

**Affiliations:** 宁波市疾病预防控制中心, 浙江省微量有毒化学物健康风险评估技术研究重点实验室, 浙江 宁波 315010; Ningbo Municipal Center for Disease Control and Prevention, Key Laboratory of Health Risk Appraisal for Trace Toxic Chemicals of Zhejiang Province, Ningbo 315010, China

**Keywords:** 固相萃取, 超高效液相色谱-串联质谱, 雌激素, 牛蛙, solid phase extraction (SPE), ultra-performance liquid chromatography-tandem mass spectrometry (UPLC-MS/MS), estrogen, bullfrog

## Abstract

建立了基于通过型固相萃取小柱净化的超高效液相色谱-三重四极杆质谱联用(UPLC-MS/MS)同时快速准确测定牛蛙中9种雌激素(雌三醇(E3)、17*β*-雌二醇(*β*-E)、17*α*-雌二醇(*α*-E)、17*α*-炔二雌醇(EE2)、雌酮(EI)、己烯雌酚(DES)、己二烯雌酚(DE)、己烷雌酚(HEX)、醋酸双烯雌酚(DD))残留的检测方法。样品经乙腈提取,经PRiME HLB固相萃取柱净化,Waters Acquity UPLC BEH C_18_柱(100 mm×2.1 mm, 1.7 μm)分离,以0.5 mmol/L氟化铵水溶液-乙腈体系为流动相梯度洗脱,流速为0.3 mL/min,采用电喷雾正负离子切换模式(ESI^+^/ESI^-^)和多反应监测(MRM)扫描方式检测,基质匹配外标法定量分析。该研究优化了液相色谱条件,相比于乙酸铵水溶液-乙腈体系和氨水溶液-乙腈体系,0.5 mmol/L氟化铵水溶液-乙腈体系作为流动相时9种雌激素普遍具有更佳的灵敏度。相比于甲醇和乙酸乙酯,乙腈作为提取溶剂时9种雌激素的提取率提高15%~40%。考察了HLB、C_18_、Silica、PRiME HLB共4种不同类型的固相萃取小柱的基质净化效应,结果表明,PRiME HLB柱具有更好的基质净化能力。经PRiME HLB净化后,所有化合物的回收率均在70%~125%之间。DD的回收率从47%提高到74%, DES的回收率从180%降低到123%,有效减弱了基质效应。在最佳的实验条件下,E3、*β*-E、*α*-E、EI、DE、HEX、DD的线性范围为0.5~100.0 μg/L, EE2和DES的线性范围为1.0~100.0 μg/L, 9种雌激素在各自的线性范围内均有良好的线性关系,相关系数为0.9953~0.9994,方法检出限为0.17~0.33 μg/kg,方法定量限为0.5~1.0 μg/kg,在2.0、10.0、80.0 μg/kg 3个加标水平下,9种雌激素的加标回收率为65.1%~128.2%,相对标准偏差为1.9%~17.6%。该方法操作简便、快速、灵敏,重复性好,可用于大批量样品的同时快速准确检测。

## Abstract (en)

Due to the harmful effects of estrogens and their prevalence in animal foods, accurate analysis of estrogen levels in animal foods is imperative in order to effectively assess food safety risks and ensure consumer safety. Therefore, a rapid and accurate method based on PRiME HLB solid phase extraction (SPE) cartridge purification and ultra-performance liquid chromatography-tandem mass spectrometry (UPLC-MS/MS) was developed to determine nine estrogen residues in bullfrogs. The nine estrogens included estriol (E3), 17*β*-estradiol (*β*-E), 17*α*-estradiol (*α*-E), 17*α*-ethinylestradiol (EE2), estrone (EI), diethylstilbestrol (DES), dienestrol (DE), hexestrol (HEX), and dienestrol diacetate (DD). This study optimized the mobile phase system, extraction solvent, and SPE cartridges. Because estrogens present weak alkalinity, adding a small amount of alkaline substance to the mobile phase benefits estrogen ionization into the ionic state, eliminates the peak trailing phenomenon, and enhances the signal response of estrogens to improve sensitivity. Estrogens have one or more hydroxyl groups in their chemical structures. According to the principle of similar solubility, polar solvents are chosen as extraction solvents. Based on the complex matrix composition of meat samples, SPE is required for purification to reduce matrix effects. The liquid chromatographic conditions were optimized, and the 0.5 mmol/L ammonium fluoride aqueous solution-acetonitrile system as mobile phases showed better sensitivity than the ammonium acetate aqueous solution-acetonitrile system and the ammonia-acetonitrile system for the nine estrogens. When acetonitrile was used as the extraction solvent, the extraction rates of all nine estrogens exceeded those of methanol and ethyl acetate and increased by 15%-40%. By focusing on the matrix purification effect of four different SPE cartridges, the results showed that the matrix purification ability of the PRiME HLB cartridge outperformed that of the HLB, C_18_, and Silica SPE cartridges. After purification by the PRiME HLB cartridge, the recoveries of all compounds were in the range of 70%-125%, and the DD recovery was increased from 47% to 74%, whereas the HEX recovery was reduced from 180% to 123%. Therefore, the PRiME HLB SPE cartridge was selected as the cleanup material for this experiment. Finally, the sample was extracted using acetonitrile, purified by PRiME HLB SPE cartridge, and separated on a Waters Acquity UPLC BEH C_18_ column (100 mm×2.1 mm, 1.7 μm) with a mobile phase of 0.5 mmol/L ammonium fluoride aqueous acetonitrile solution at a flow rate of 0.3 mL/min. The detection was conducted in positive and negative ion switching mode (ESI^+^/ESI^-^) and multiple reaction monitoring (MRM) scanning, and it was quantified using a matrix-matched external standard method. Under the optimal experimental conditions, the linear ranges were 0.5-100.0 μg/L for E3, *β*-E, *α*-E, EI, DE, HEX, and DD, and 1.0-100.0 μg/L for EE2 and DES. The nine estrogens showed good linearity in all linear ranges, with correlation coefficients of 0.9953-0.9994. The limits of detection were 0.17-0.33 μg/kg, and the limits of quantification were 0.5-1.0 μg/kg. The recoveries of the nine estrogens spiked at the three spiked levels of low (2.0 μg/kg), medium (10.0 μg/kg), and high (80.0 μg/kg) were 107.4%-125.3%, 67.0%-123.3%, and 65.1%-128.2%, respectively. The relative standard deviations were 1.9%-17.6%. The method established in this study was applied to detect nine estrogen residues in 50 commercially available bullfrog samples, and the results showed that HEX, EI, and DES were detected in few samples. The method is simple, rapid, sensitive, and reproducible, and can be used for the simultaneous, rapid and accurate determination of large quantities of samples.

牛蛙属于大型的食用蛙类,近年已成为我国养殖产量最大的两栖动物,逐渐成为人们餐桌上的美食。牛蛙中的雌激素含量是人们普遍关注的食品安全性问题之一。雌激素主要有天然雌激素(雌三醇、雌酮、雌二醇等)和人工合成雌激素(17*α*-炔二雌醇、己烯雌酚、己二烯雌酚、己烷雌酚、醋酸双烯雌酚等),天然雌激素广泛存在于动物体内,但由于人工合成雌激素具有影响动物性别分化、缩短动物生长周期和增加脂肪沉积等效应,常被不法养殖户加入饲料中提高饲养效率,造成其在动物体内残留^[1⇓-3]^。雌激素不易降解,通过食物链进入人体后,在极低的含量下(1.0 ng/L)就会对生物体产生明显的影响^[4]^,具有较强的生物活性和潜在的致癌性^[2,3]^,可能会引发先兆子痫^[5]^、肝癌(肝纤维化)^[6]^、乳腺癌^[7,8]^等疾病,在农业农村部发布的《食品动物中禁止使用的药品及其他化合物清单》(中华人民共和国农业农村部公告第250号)^[9]^中规定食品动物禁止使用己二烯雌酚、己烯雌酚、己烷雌酚及其盐、酯。鉴于雌激素的危害性以及在动物性食品中的普遍存在,建立灵敏度高、准确性好、操作简便的动物性食品中雌激素残留的分析方法十分必要。

动物性食品中雌激素的常用检测方法主要有气相色谱-串联质谱法(GC-MS/MS)^[10⇓-12]^和液相色谱-串联质谱法(LC-MS/MS)^[13⇓⇓⇓⇓-18]^。GC-MS/MS要求目标分析物具有一定的挥发性和热稳定性,雌激素对热稳定,但不易挥发,需要对其进行衍生化处理以增加挥发性,衍生步骤繁琐且难以控制,同时需寻找特异性的衍生化试剂^[10⇓-12]^,对于大批量的样品检测耗时长、效率低。而LC-MS/MS则无需进行衍生,能够实现高通量快速检测,且假阳性率低,适用于食品基质中痕量药物残留的分析,是目前主要的检测方法之一^[13⇓⇓⇓⇓⇓⇓-20]^。肉类食品基质成分复杂,需采用预处理技术进行净化以减少基质效应(ME),常用的技术有固相萃取(SPE)、分散固相萃取、固相微萃取、QuEChERS等^[17⇓⇓⇓⇓⇓-23]^,一般来说,SPE的重复性和精密度要优于其他3种萃取技术,更适用于肉类食品中痕量雌激素残留的准确定量检测。文献^[20⇓-22]^多数采用吸附性SPE小柱,但该类小柱需经活化、上样、淋洗和洗脱4个程序,操作繁琐、费时,PRiME HLB通过型SPE小柱大大提高了检测的便捷性,适用于大样本的高通量快速筛查,在肉类食品的残留分析中已有较多的应用^[24⇓-26]^,但未见肉类雌激素测定的应用文献报道。另外,在同时分析雌激素的多残留时,色谱流动相改性剂的种类和浓度对雌激素分析灵敏度有显著影响,文献报道较多的有低浓度氨水^[1,18]^和低浓度氟化铵^[23]^,并且认为添加氟化铵可以提高类固醇激素的电喷雾离子化效率和响应值。本研究通过优化流动相体系,以0.5 mmol/L氟化铵水溶液-乙腈为流动相,采用PRiME HLB通过型SPE小柱净化牛蛙中的9种雌激素,采用超高效液相色谱-三重四极杆质谱联用法(UPLC-MS/MS)建立了快速、灵敏、准确的分析方法,为动物食品中雌激素残留的准确测定提供了新的思路。

## 1 实验部分

### 1.1 仪器、试剂与材料

Exion LC-TRIPLE QUAD 6500+超高效液相色谱-三重四极杆质谱仪(美国AB Sciex); N-EVAP 112水浴干浴氮吹仪(美国Organomation公司); Sigma 3-30K高速台式冷冻离心机(德国Sigma公司); Multi Reax振荡器(德国Heidolph公司); 20位固相萃取装置(美国Agilent公司)。

乙腈和甲醇(HPLC级)均购自美国Thermo Fisher公司;乙酸乙酯(HPLC级)购自美国TEDIA公司;乙酸铵、氨水(HPLC级)购自德国Merck公司;氟化铵(纯度≥99.99%)购自麦克林公司;有证标准品雌三醇(estriol,E3)、17*β*-雌二醇(17*β*-estradiol,*β*-E)、17*α*-雌二醇(17*α*-estradiol,*α*-E)、17*α*-炔二雌醇(17*α*-ethynylestradiol,EE2)、雌酮(estrone,EI)、己烯雌酚(diethylstilbestrol,DES)、己二烯雌酚(dienestrol,DE)、己烷雌酚(hexestrol,HEX)、醋酸双烯雌酚(dienestrol diacetate,DD)均购自北京振翔科技有限公司,纯度均大于99%。

PRiME HLB(200 mg/6 mL)、HLB(200 mg/6 mL)、Silica(500 mg/6 mL)固相萃取柱均购自美国Waters公司,C_18_(500 mg/6 mL)固相萃取柱购自美国Supelco公司。

50份牛蛙样品购自宁波当地菜市场和超市。

### 1.2 样品前处理

取1.0 g样品于50 mL离心管中,加入5 mL乙腈涡旋提取5 min, 8000 r/min冷冻离心5 min,取上清液,直接过PRiME HLB柱。收集流出液,氮吹至干,最后用50%(v/v)乙腈水溶液定容至1.0 mL,过0.22 μm聚四氟乙烯滤膜,UPLC-MS/MS检测。

### 1.3 溶液配制

标准溶液:分别准确称取9种雌激素标准品1.0 mg于9个10 mL棕色容量瓶中,用甲醇溶解并定容至刻度,配成质量浓度为100.0 mg/L的标准储备液,于-20 ℃避光保存。分别准确吸取上述9种标准储备液1.0 mL于10 mL棕色容量瓶中,用甲醇定容至刻度,得质量浓度均为10.0 mg/L的9种混合标准中间溶液。

系列标准工作溶液:取适量10.0 mg/L的9种混合标准中间溶液,用50%(v/v)乙腈水溶液稀释定容,制得质量浓度分别为0.5、1.0、2.0、5.0、10.0、20.0、50.0、100.0 μg/L的系列标准工作溶液。

基质匹配标准工作溶液:取1.0 g空白样品,按1.2节步骤进行前处理,得到空白基质溶液,再分别加入适量的10.0 mg/L 9种混合标准中间溶液,然后再用50%(v/v)乙腈水溶液稀释定容,制得质量浓度分别为0.5、1.0、2.0、5.0、l0.0、20.0、50.0、100.0 μg/L的系列基质匹配标准工作溶液。

### 1.4 色谱-质谱条件

色谱条件 色谱柱为Waters Acquity UPLC BEH C_18_柱(100 mm×2.1 mm, 1.7 μm);流动相A为0.5 mmol/L氟化铵水溶液,流动相B为乙腈,流量为0.3 mL/min,柱温35 ℃,进样体积为2 μL。梯度洗脱:0~0.5 min, 3%B; 0.5~2.0 min, 3%B~40%B; 2.0~8.0 min, 40%B~95%B; 8.0~10.9 min, 95%B; 10.9~11.0 min, 95%B~3%B; 11.0~12.0 min, 3%B。

质谱条件 雾化器压力:344.7 kPa (50 psi);辅助器压力:344.7 kPa (50 psi);气帘气压力:241.3 kPa (35 psi);电喷雾电压:+/- 4500 V;离子源温度:500 ℃;检测方式:电喷雾正负离子切换模式(ESI^+^/ESI^-^),多反应监测(MRM)。其他质谱参数见表1。

**表 1:** 9种目标化合物的质谱参数

Compound	t_R_/min	Precursor ion (m/z)	Product ion (m/z)	Declustering potential/V	Collision energy/eV
Estriol (E3)	3.37	287.3	171.1^*^	-100	-47
			145.2	-100	-44
17β-Estradiol (β-E)	4.49	271.4	145.2^*^	-60	-65
			183.1	-80	-54
17α-Estradiol (α-E)	4.71	271.4	145.2^*^	-60	-53
			183.1	-80	-54
17α-Ethynylestradiol (EE2)	4.78	295.2	145.2^*^	-80	-50
			159.2	-80	-35
Estrone (EI)	4.93	269.4	145.4^*^	-60	-41
			159.2	-80	-41
Diethylstilbestrol (DES)	5.09	267.1	237.3^*^	-100	-28
			222.2	-100	-33
Dienestrol (DE)	5.23	265.2	93.0^*^	-80	-31
			171.1	-80	-25
Hexestrol (HEX)	5.23	269.5	119.1^*^	-80	-41
			133.9	-80	-16
Dienestrol diacetate (DD)	7.23	351.0	237.2^*^	60	30
			173.2	60	36

* Quantitative ion.

## 2结果与讨论

### 2.1 流动相的优化

文献^[3]^报道,水-乙腈作为流动相比水-甲醇更有利于激素的离子化,本研究选用水-乙腈体系进行进一步优化,着重考察了不同浓度及种类的流动相改性剂(如乙酸铵、氨水和氟化铵)加入到水-乙腈体系作为混合流动相对9种雌激素的分离效果和灵敏度的影响。结果表明,使用不同浓度的乙酸铵水溶液-乙腈体系,目标化合物灵敏度均较差,且雌醇类化合物在20.0 μg/L质量浓度下不能被检出,不能满足痕量检测的要求。使用0.1%(v/v)氨水溶液-乙腈体系,负离子模式下检测化合物的灵敏度均有了较明显提高,可见NH_3_结合H的能力更强,使其酚羟基上的H脱去呈负离子,有利于雌激素电离成离子状态,增强其信号响应,提高灵敏度。但实验结果显示,雌醇类化合物的信号强度较雌酚类化合物弱,可能是因为HEX、DES和DE均含有两个酚羟基,而雌醇类和雌酮只有一个酚羟基。采用0.5 mmol/L氟化铵水溶液-乙腈体系,9种雌激素的灵敏度(以信号强度计)均提高了一个数量级,结果(以HEX为例)见图1, 20.0 μg/L的HEX标准溶液在10 mmol/L乙酸铵水溶液-乙腈体系、0.1%(v/v)氨水溶液-乙腈体系和0.5 mmol/L氟化铵水溶液-乙腈体系作为流动相条件下,目标化合物的灵敏度(以信号强度计)分别为10^3^、10^4^和10^5^,说明氟化铵可在电喷雾负离子模式下为雌激素类化合物提供更好的信号,这可能是因为氟化铵中的F电负性较强,更易与H结合。而对于醋酸双烯雌酚,氟化铵在水溶液中成弱酸性,更利于其得到H变成[M+H]^+^在正离子模式下检测。

**图 1:**
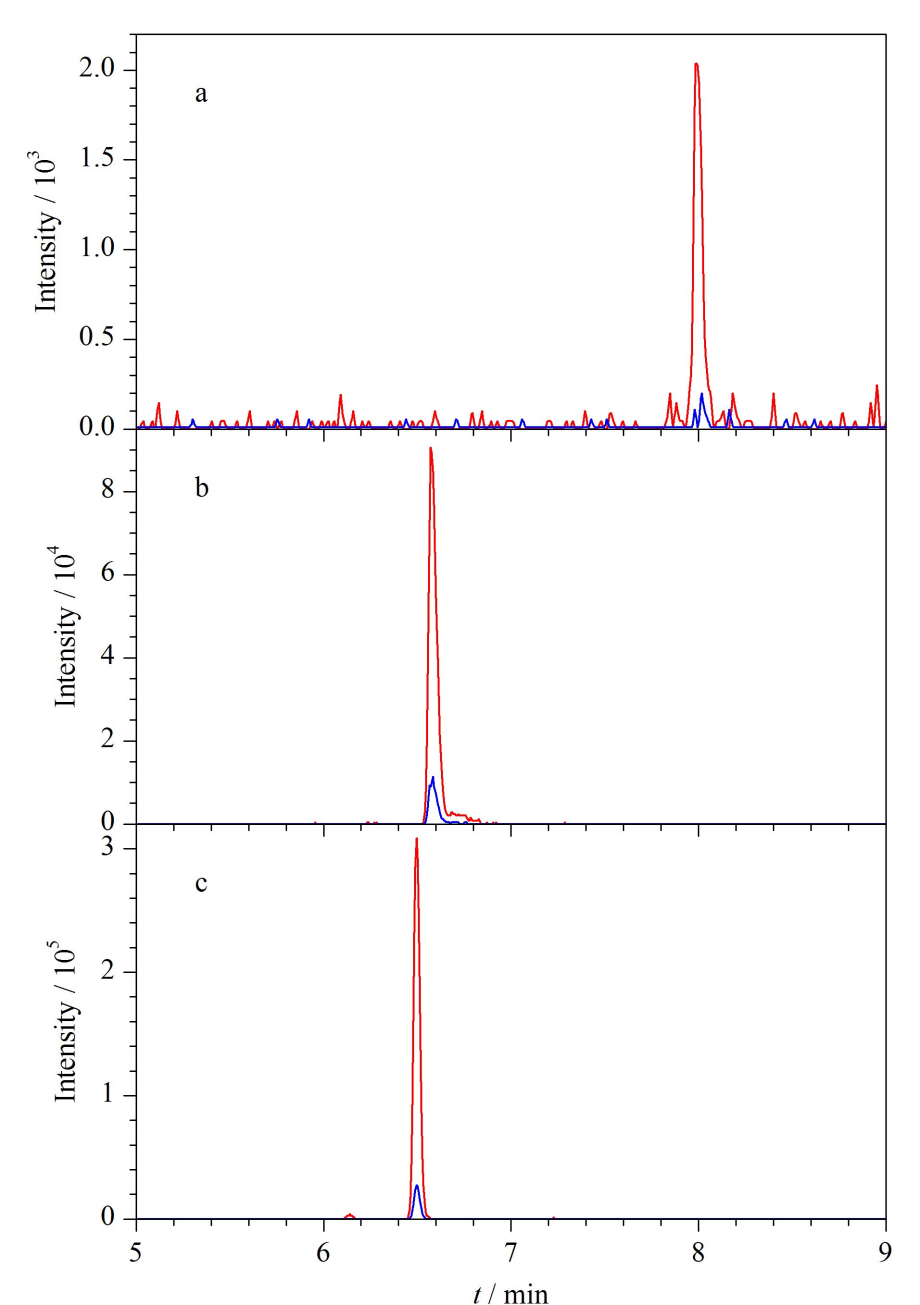
HEX在不同流动相体系下的MRM图

### 2.2 提取溶剂的筛选

DD结构式中没有羟基但带有酯基,其他8种雌激素的化学结构中均带有一个或多个羟基基团,根据相似相溶原理,其在极性溶剂中的溶解度应较为理想,本实验根据文献考察了甲醇^[18]^、乙腈^[13]^和乙酸乙酯^[27]^ 3种溶剂对9种目标化合物的提取效率影响。实验结果见图2。结果表明,当提取溶剂依次为乙腈、乙酸乙酯、甲醇时,9种目标化合物中提取效率为70%~110%的数量逐渐减少。当乙腈作为提取溶剂时,9种雌激素的提取率均高于甲醇和乙酸乙酯,且提高15%~40%。结果表明乙腈作为提取溶剂优于甲醇和乙酸乙酯。因此,本实验选择乙腈作为牛蛙中雌激素的提取溶剂。

**图 2:**
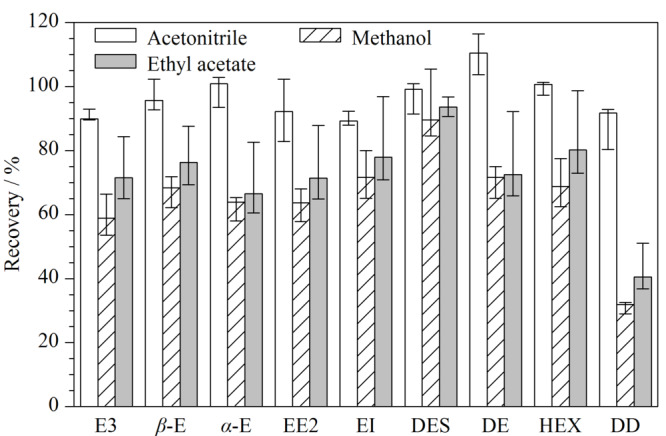
不同溶剂的提取效率对比(*n*=3)

### 2.3 固相萃取柱的优化

根据文献^[20⇓-22]^, HLB、C_18_、Silica固相萃取柱被应用于类固醇化合物的基质净化中。PRiME HLB通过型SPE小柱相较这3种SPE小柱,可大幅减少操作时间,有效提高检测效率。因此,本研究重点考察了HLB、C_18_、Silica和PRiME HLB柱4种不同的SPE小柱对牛蛙中9种雌激素残留检测的基质净化影响。净化过程如下。

HLB SPE柱:上清液氮吹至干,用3 mL 20%(v/v)乙腈水溶液溶解,过预先活化好的HLB柱,3 mL水淋洗,3 mL乙腈-甲醇(1:1, v/v)洗脱;C_18_ SPE柱:上清液氮吹至干,用3 mL (v/v)乙腈水溶液溶解,过预先活化好的C_18_柱,3 mL水淋洗,3 mL乙腈洗脱;Silica SPE柱:上清液氮吹至干,用3 mL (v/v)乙腈水溶液溶解,过预先活化好的Silica柱,3 mL正己烷淋洗,3 mL乙腈洗脱;PRiME HLB SPE柱:上清液直接过PRiME HLB柱。分别收集流出液,氮吹至干,最后用50%(v/v)乙腈水溶液定容至1.0 mL,过0.22 μm聚四氟乙烯滤膜,UPLC-MS/MS检测。比较4种SPE小柱净化后牛蛙中9种雌激素的加标回收率,结果见图3和图4。

**图 3:**
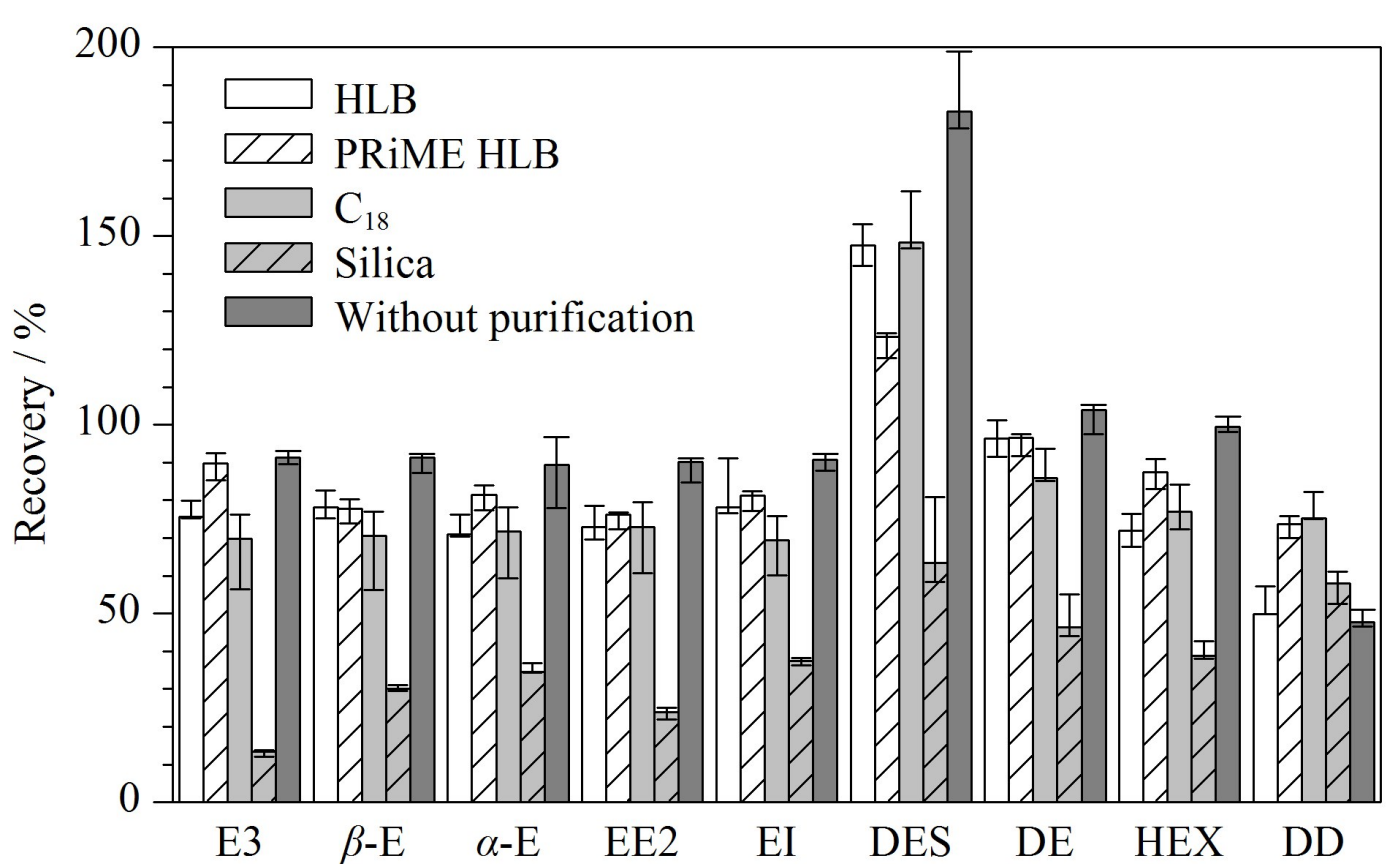
不同固相萃取柱的净化性能对比(*n*=3)

**图 4:**
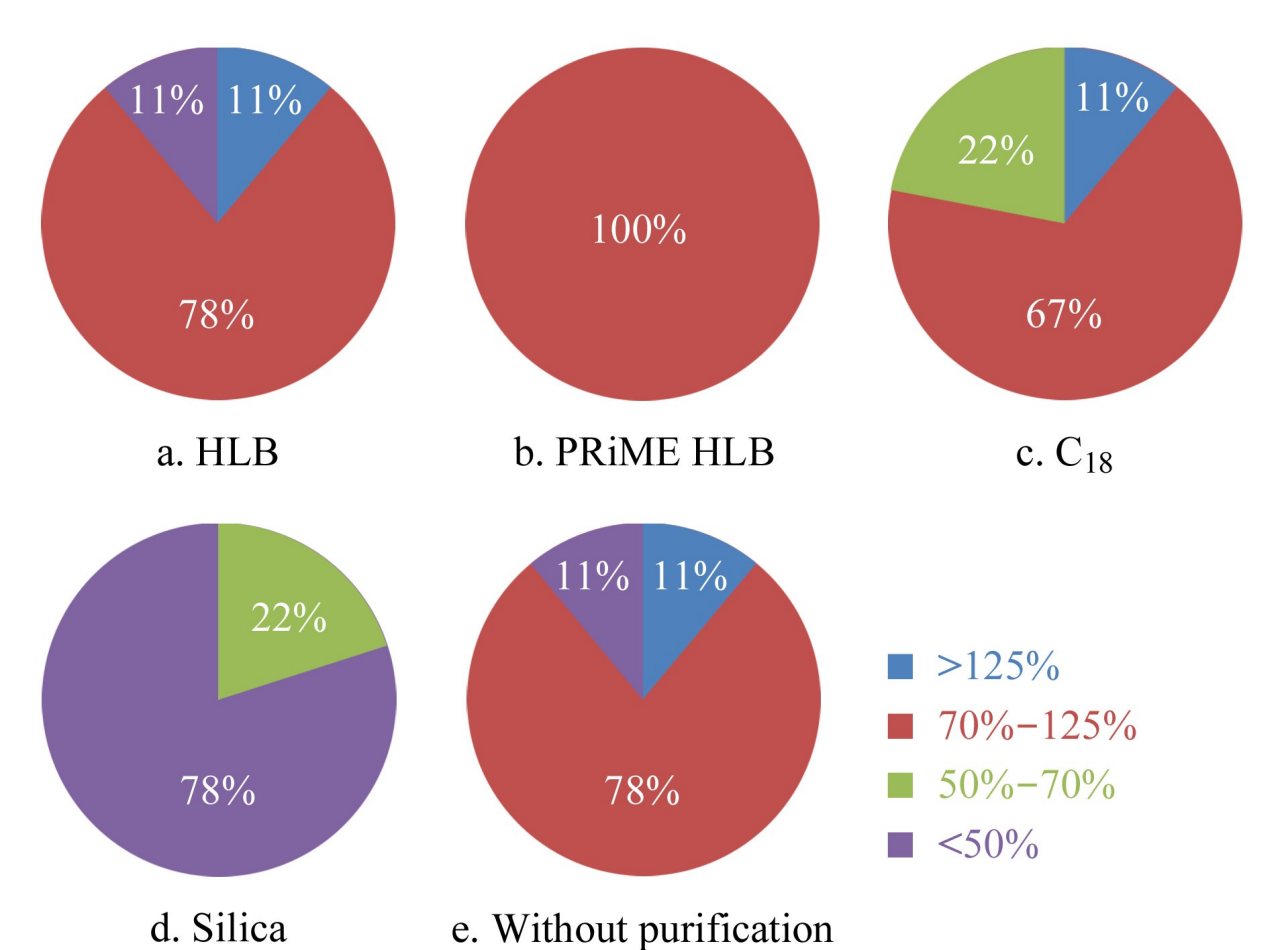
不同固相萃取柱净化时9种雌激素的加标回收率占比

由图3和图4可知,未经净化处理的牛蛙样品,9种目标化合物的回收率在70%~125%之间的占比为77.8%, DES存在较强的基质增强效应,DD存在较强的基质抑制效应,两个化合物的回收率不能满足检测要求。经Silica固相萃取柱净化后,DD的回收率略有提升,但其他雌激素的回收率下降明显,9种目标化合物回收率均低于70%,可见硅胶对雌激素的吸附保留作用弱,不适合用做雌激素的净化材料。经HLB固相萃取柱净化后,DD的回收率略有提高,DES的回收率下降了35%,但是其他雌激素的回收率反而略有下降,通过对比实验发现HLB小柱对DES、DE、HEX这3种雌激素的吸附力较强,单纯用乙腈无法洗脱,必须用乙腈-甲醇(1:1, v/v)才可以洗脱,可能HLB小柱对雌激素吸附能力较强无法被完全洗脱,导致样品净化过程中目标分析物流失。经C_18_固相萃取柱净化后,DD的回收率有了明显提升,回收率提高30%, DES的回收率下降35%,可见C_18_柱可改善DE的基质增强效应和DD的基质抑制效应,但其他雌激素的回收率也略有不同程度的下降,使得回收率在70%~125%之间的化合物占比未提高。经PRiME HLB柱净化后,虽然部分化合物的回收率略微下降,但是所有化合物的回收率均在70%~125%之间,且DD的回收率从47%提高到了74%,有效改善了其基质抑制效应,同时,DES的回收率从180%降低到了123%,有效降低了其基质增强效应。因此,本实验选择PRiME HLB固相萃取柱作为净化材料。

### 2.4 基质效应考察

ME=(基质匹配工作溶液中各目标物的峰面积-标准工作溶液中各目标物的峰面积)/标准工作溶液中各目标物的峰面积。ME的正负分别表示基质增强效应和基质抑制效应。当│ME│小于20%时,表示弱基质效应,当│ME│为20%~50%时,表示中等基质效应,当│ME│大于50%时,表示强基质效应。空白样品经提取后,加标可得10 μg/L的基质匹配工作溶液,将其与同浓度的标准工作溶液分别进行UPLC-MS/MS检测,计算基质效应。结果表明,77.8%的化合物表现出弱基质效应,且均为弱基质抑制效应。DES和DD表现出强基质效应,其中DES的ME为63.9%,表现出强基质增强效应,DD的ME为-67.4%,表现出强基质抑制效应。经PRiME HLB固相萃取柱净化后,计算固相萃取后的基质效应(见图5),结果表明,DES的ME为39.8%, DD的ME为-25.7%,其强基质效应有了明显减弱,但还是存在中等基质效应,因此本实验采用基质匹配标准曲线,以降低基质效应对目标化合物的影响。

**图 5:**
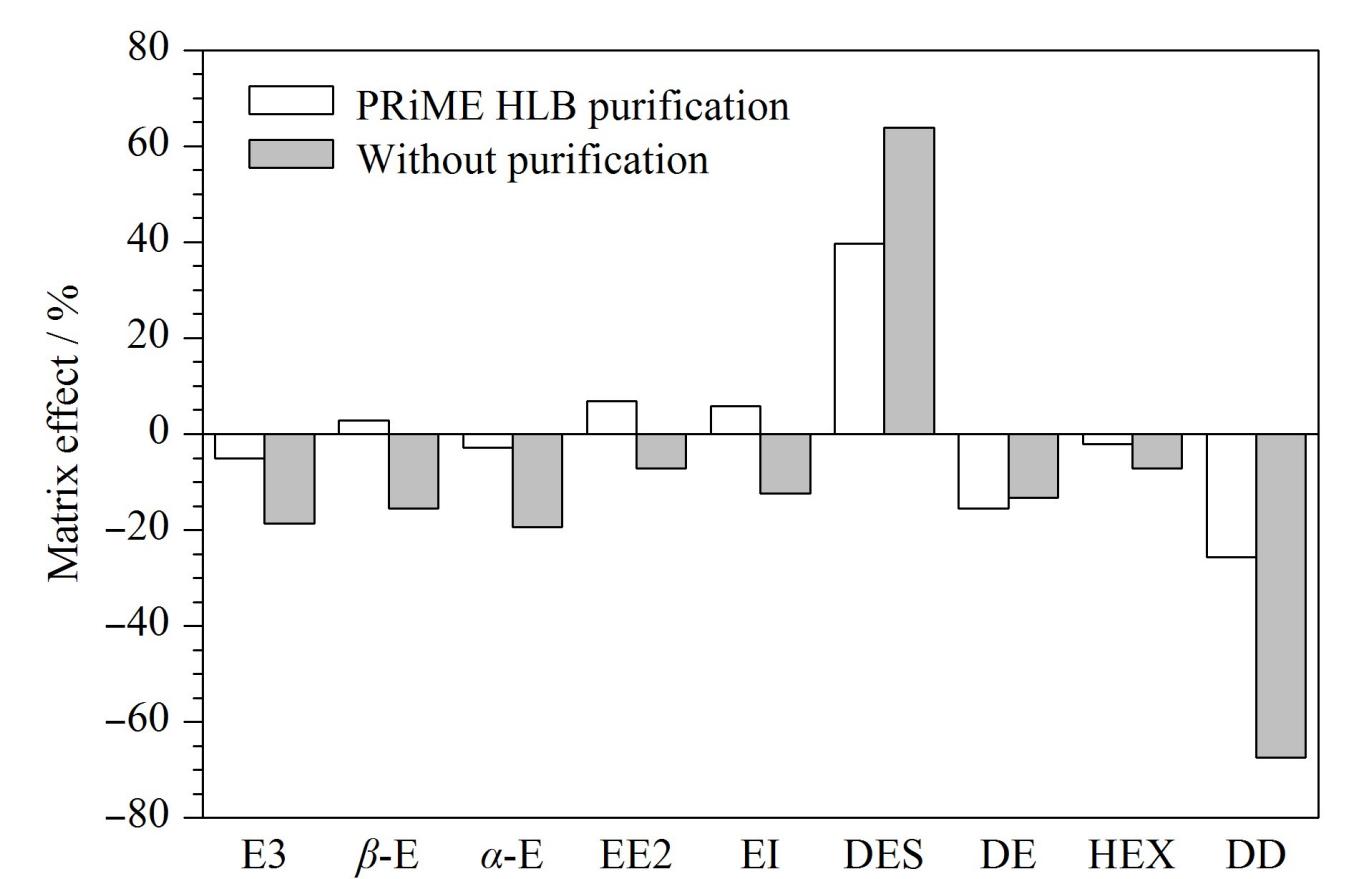
牛蛙中9种雌激素在净化前后的基质效应

### 2.5 方法学考察

#### 2.5.1 线性关系、检出限和定量限

测定1.3节配制的系列基质匹配混合标准溶液,以各个雌激素的峰面积为纵坐标,质量浓度为横坐标,考察9种雌激素的线性关系。结果显示,9种雌激素在相应的浓度范围内具有良好的线性关系,相关系数(*R*)≥0.9953 (见表2)。采用空白样品加标的方式确定9种雌激素的方法检出限(*S/N*=3)为0.17~0.33 μg/kg,方法定量限(*S/N*=10)为0.5~1.0 μg/kg(见表2)。质量浓度为10 μg/L的9种目标化合物的MRM图见图6。

**表 2:** 9种化合物的线性方程、相关系数、检出限、定量限、加标回收率和精密度(*n*=6)

Compound	Linear equation	R	LOD/ (μg/kg)	LOQ/ (μg/kg)	Linear range/(μg/L)	Recoveries and RSDs at three spiked levels/%								
						2.0 μg/kg			10.0 μg/kg		80.0 μg/kg	
						Rec.	RSD		Rec.	RSD	Rec.	RSD
E3	y=24464x-14627	0.9993	0.17	0.5	0.5-100.0	110.5	4.9		86.6	5.8	83.8	4.8
β-E	y=18929x-7817	0.9994	0.17	0.5	0.5-100.0	107.4	5.1		85.3	3.6	84.4	8.9
α-E	y=29330x-14970	0.9988	0.17	0.5	0.5-100.0	110.6	5.4		85.0	6.2	85.4	6.7
EE2	y=15889x-6086	0.9988	0.33	1.0	1.0-100.0	108.7	7.0		85.0	5.4	87.7	7.7
EI	y=32545x-15341	0.9992	0.17	0.5	0.5-100.0	112.4	4.9		89.4	1.9	89.0	8.7
DES	y=18623x-21510	0.9953	0.33	1.0	1.0-100.0	125.3	10.5		123.3	6.4	128.2	8.6
DE	y=97509x-59171	0.9977	0.17	0.5	0.5-100.0	123.1	2.8		105.5	6.5	107.0	5.3
HEX	y=43155x-21568	0.9987	0.17	0.5	0.5-100.0	115.2	4.9		86.8	6.9	87.7	7.9
DD	y=96643x-50141	0.9987	0.17	0.5	0.5-100.0	117.1	11.3		67.0	5.9	65.1	17.6

*y*: peak area; *x*: mass concentration, μg/L; Rec.: recovery.

**图 6:**
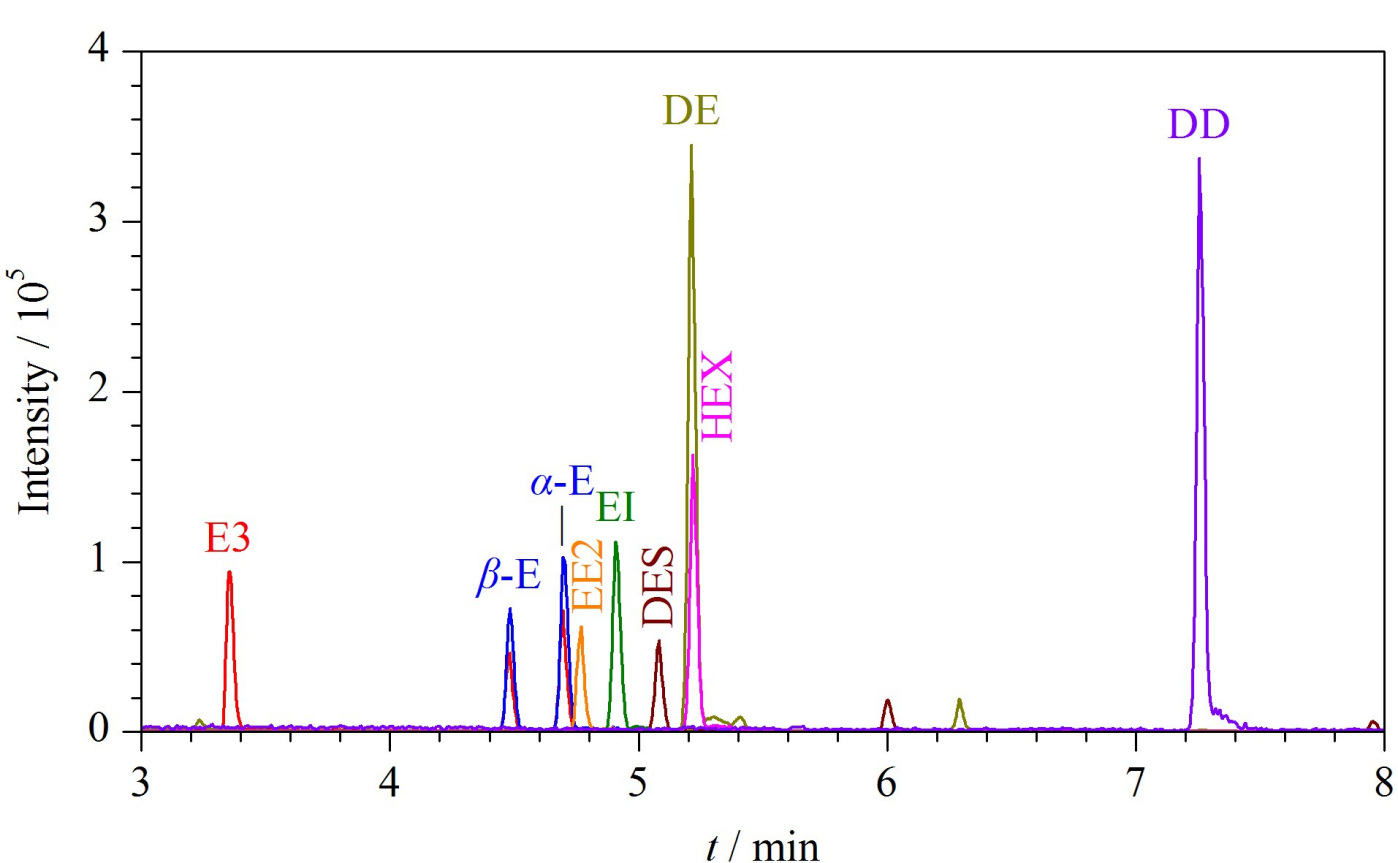
9种目标化合物的MRM图

#### 2.5.2 加标回收率和精密度

以空白牛蛙样品为基质,添加低(2.0 μg/kg)、中(10.0 μg/kg)、高(80.0 μg/kg) 3个水平的混合标准溶液,按1.2节步骤进行样品前处理,采用UPLC-MS/MS检测。每个加标水平做6次平行实验,结果显示,9种雌激素在低、中、高3个加标水平下的回收率(*n*=6)分别为107.4%~125.3%、67.0%~123.3%、65.1%~128.2%, RSD(*n*=6)分别为2.8%~11.3%、1.9%~6.9%、4.8%~17.6%,见表2。其中DES在3个水平下的加标回收率均较高,可能原因是基质对其增强作用比较显著,其线性方程的相关系数较其他雌激素低,且检出限也较高,一定程度说明基质效应对DES影响较大;中高浓度DD的加标回收率较低,可能原因是正离子监测模式下,弱酸性的氟化铵提供H^+^的能力在高浓度系列下优势不那么明显。

### 2.6 样品测定

应用本研究建立的方法对市售50份牛蛙样品中的9种雌激素残留进行检测,结果表明,有HEX、EI、DE检出的样品份数分别为9、6、3份,其中有3份样品同时检测出HEX、EI、DE,有3份样品同时检测出HEX、EI,有3份样品只检测出HEX。HEX、EI、DE三者的检出量分别为0.5~0.8、0.5~0.7、0.6~0.8 μg/kg,含量较低,均接近定量限。

## 3 结论

本文通过筛选提取溶剂,优化流动相体系,对比研究HLB、C_18_、Silica、PRiME HLB等4种不同类型固相萃取小柱的基质净化效应,建立了基于PRiME HLB通过型固相萃取-超高效液相色谱-串联质谱同时测定牛蛙中9种雌激素的分析方法,并将方法应用于50份市售牛蛙样品中雌激素含量的检测。建立的SPE-UPLC-MS/MS同时测定牛蛙中9种雌激素残留的分析方法,具有操作简便、高效、准确、灵敏的特点,适合于实验室大批量样品的快速检测。
